# Corneal Cell Adhesion to Contact Lens Hydrogel Materials Enhanced via Tear Film Protein Deposition

**DOI:** 10.1371/journal.pone.0105512

**Published:** 2014-08-21

**Authors:** Claire M. Elkins, Qin M. Qi, Gerald G. Fuller

**Affiliations:** Department of Chemical Engineering, Stanford University, Stanford, California, United States of America; Seoul National University, Republic of Korea

## Abstract

Tear film protein deposition on contact lens hydrogels has been well characterized from the perspective of bacterial adhesion and viability. However, the effect of protein deposition on lens interactions with the corneal epithelium remains largely unexplored. The current study employs a live cell rheometer to quantify human corneal epithelial cell adhesion to soft contact lenses fouled with the tear film protein lysozyme. PureVision balafilcon A and AirOptix lotrafilcon B lenses were soaked for five days in either phosphate buffered saline (PBS), borate buffered saline (BBS), or Sensitive Eyes Plus Saline Solution (Sensitive Eyes), either pure or in the presence of lysozyme. Treated contact lenses were then contacted to a live monolayer of corneal epithelial cells for two hours, after which the contact lens was sheared laterally. The apparent cell monolayer relaxation modulus was then used to quantify the extent of cell adhesion to the contact lens surface. For both lens types, lysozyme increased corneal cell adhesion to the contact lens, with the apparent cell monolayer relaxation modulus increasing up to an order of magnitude in the presence of protein. The magnitude of this increase depended on the identity of the soaking solution: lenses soaked in borate-buffered solutions (BBS, Sensitive Eyes) exhibited a much greater increase in cell attachment upon protein addition than those soaked in PBS. Significantly, all measurements were conducted while subjecting the cells to moderate surface pressures and shear rates, similar to those experienced by corneal cells *in vivo*.

## Introduction

The tear film is a complex, multilayered structure composed of lipids, mucins, and water-soluble proteins that perform a wide range of functions, from maintaining tear film mechanical stability [1], [2] to preventing microbial infection [3]. The aqueous layer of the tear film contains several proteins which are known to deposit onto contact lenses during wear, greatly affecting the adhesion and viability of bacteria on the lens surface [4], [5]. In particular, although antimicrobial tear-film proteins such as lactoferrin and lysozyme have been shown to increase the total number (both viable and nonviable) of bacteria that adhere to the lens, they have also been shown to decrease the viability of certain bacteria [6]. The observed increase in adhesion presumably occurs because the deposited protein presents functional moieties that promote formation of bacteria-lens attachments [7], [8]. The effects on bacterial viability are less well understood but may depend on the relationship between the conformation of the adsorped protein and its mode of antibacterial action [9], [10].

In spite of an abundance of research on tear film protein interactions with bacteria, there is relatively little information available about the interaction between tear film protein deposits and the corneal surface itself (i.e., corneal epithelial cells). This is an important field of inquiry, as the contact lens remains in intimate contact with the cornea for a prolonged period of time. Previous studies have shown that epithelial barrier permeability increases after extended soft contact lens wear [11], [12]. If protein accumulation on the lens over time serves to increase corneal cell attachment, this could impact overall corneal health and lens comfort. Therefore the aim of the current study was to develop a method whereby corneal epithelial cell adhesion to commercial contact lens surfaces could be directly measured. This technique was used to quantify the effect of protein deposition on corneal cell adhesion for two commercially available lenses. Although the corneal epithelial cell monolayers tested in this study are not identical to the apical corneal layer in a fully differentiated multilayer corneal model, we believe the methods and results of the present study serve as a closely related, well-characterized model system that provides insight into potential mechanical interactions between corneal epithelial cells and contact lens hydrogels in the presence of these proteins.

The tear film contains antibacterial tear proteins (lysozyme, lactoferrin, albumin, among others) that are known to play a role in preventing infection and inflammation of the eye surface [3], [13]. During contact lens wear, these components accumulate on the lens surface, forming a protein-rich conditioning film [14]–[16]. The composition of this film (ratio and conformation of deposited proteins, etc.) depends on the chemical composition of the contact lens [16]–[19]. The current work studied the impact of protein accumulation on two silicone hydrogel lenses, PureVision balafilcon A (PV) and AirOptix lotrafilcon B (AO), both of which have been shown to take up deposits of lysozyme and lactoferrin in a way that increases the adhesion of certain bacteria [6].

Although the impact of the protein conditioning film on corneal epithelial health has not been extensively studied, the impact of different combinations of silicon hydrogel lenses and multipurpose care solutions on corneal health has been explored previously [11], [20]. These studies have found that varying the contact lens type and care solution can strongly affect epithelial barrier function as measured by the extent of corneal staining observed after lens wear [12], [21], [22]. It would be invaluable to explore another potential impact of lens-care solution interaction, namely whether different care solutions result in varying degrees of cell adhesion to the contact lens surface. Therefore, in addition to measuring the effect of deposited protein, the present study also examined the influence of three different lens soaking solutions: (phosphate buffered saline (PBS), borate buffered saline (BBS), and Sensitive Eyes Plus Saline Solution (Sensitive Eyes)), on corneal cell adhesion to the lens surface.

Live cell interactions with a contact lens surface have been investigated previously via the use of a small tribological testing pin covered with a cut-out section of contact lens material [23]. In these studies, the pin was oscillated at 1.2 kHz with a contact pressure of ∼12 kPa against the surface of a corneal epithelial cell monolayer in order to determine a friction coefficient [23]. The current study is differentiated from that work in several ways. Most notably, a two-hour period of physical contact prior to shearing the contact lens allowed corneal cells the opportunity to form focal adhesions with the contact lens surface. In addition, the present work involved exposing the monolayer to a relatively large ∼2.8 mm^2^ contact area with minimal normal force, resulting in a moderate contact pressure of ∼1 kPa, less than that estimated for an eyelid during a normal eyeblink (3.5–4.0 kPa) [24]–[26]. Finally, the top lens motion was executed in a step-wise fashion once every ten minutes, rather than at a high oscillatory frequency. The contact lens sliding speed was ∼0.1 cm/s, below that estimated for blinking speed, 12 cm/s [24], [26]. In this way, the present study was able to analyze the extent of corneal cell attachment to contact lenses without exceeding the moderate contact pressure and sliding speed thought to exist *in vivo*.

## Methods

### The Linear Cell Monolayer Rheometer

**Figure 1:**
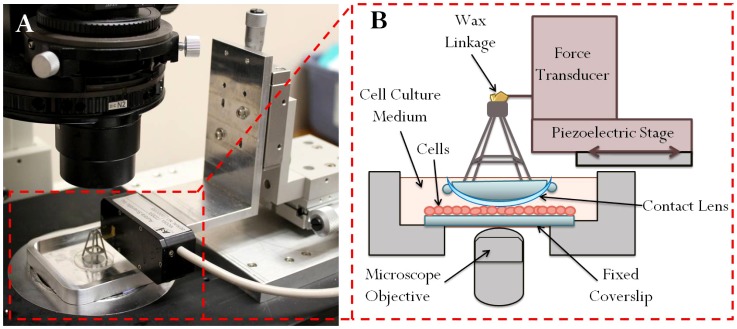
Photograph (A) and schematic (B) of the LCMR instrument mounted on a microscope.

In order to quantify the adhesion of corneal epithelial cells to the contact lens surface, a modified version of a linear cell monolayer rheometer (LCMR) was used. Figure 1 provides a diagram of this instrument. The LCMR instrument is centered around a metal dish with a glass coverslip mounted in the base. Human telemorase-immortalized corneal epithelial cells (hTCEpi) [27] were kindly provided as a gift from the Suzanne Fleiszig lab of the University of California, Berkeley. htCEpi cells were cultured in the LCMR dish prior to each experiment; details of substrate preparation and cell culture are provided in a later section. At the outset of each experiment, the dish containing an 85–95% confluent cell monolayer covered by a layer of cell culture medium was mounted onto an inverted microscope (Nikon Eclipse T*i*, Nikon, Melville, NY). A viewing port beneath the coverslip allowed optical observation of the cells, both to verify cell density as well as to monitor cell deformation during the experiment. An objective heater (Bioptechs Objective Heater System, Bioptechs, Butler, PA) attached to a 100X oil immersion objective (CFI Plan Apo VC 100X Oil, Nikon) maintained the coverslip surface at 37°C.

The upper portion of the LCMR apparatus consisted of a contact lens mounted to a holder. The pretreatment and mounting of the contact lenses is described in a later section. At the start of each experiment, the mounted contact lens was descended through the cell culture media until contact was made with the hTCEpi cells, as verified by observation of an approximately 10% increase in cell diameter through the microscope. The gap between the bottom coverslip and the contact lens surface was determined by analyzing the adjustment required to change the plane of focus from 1 *µ*m beads present at low density on the bottom plate to 1 *µ*m beads present at low density on the contact lens. This focus adjustment was correlated to gap distance using a calibration curve created using spacer beads of known diameter from 2 to 15 *µ*m. The gap distance was generally ∼5 *µ*m, approximately the height of an hTCEpi cell monolayer. This gap distance was remeasured both during and after the experiment and was found to remain constant throughout the course of the experiment. No additional normal force was placed on the cells beyond that caused by the mass of the contact lens and mount. Dividing this force by the cell contact area (determined by examining the furthest points in the field of view where cells were compressed), it was possible to estimate the maximum contact pressure experienced by the cells as ∼1 kPa, slightly lower than that predicted for eyelid pressure during a normal eyeblink (3.5–4.0 kPa) [24]–[26].

After the contact lens made contact with the cell layer, the top of the mounting apparatus was affixed to a force transducer tip with a small amount of melted wax. This wax then solidified at room temperature to form a rigid linkage between the force transducer tip and the contact lens mount. After the wax hardened, the force transducer output was zeroed to establish a baseline for the unstrained system. The force transducer (400A, Aurora Scientific, Aurora, ON, Canada) was mounted on a computer-controlled piezoelectric stage (M-011PS, Physik Instrumente, Karlsruche, Germany) programmed to induce lateral displacement of the top lens, thereby shearing the top lens against the cell monolayer. The top plate was left in contact with the cell layer for two hours prior to any further mechanical deformation, in order to allow any cell-lens attachments to form. The two-hour waiting period was chosen because mechanical measurements revealed that beyond two hours no further increase in cell attachment was observed (i.e., mechanical data taken after two hours was not significantly different from that collected after 12 hours). After the two hour waiting period, the stage executed a series of step strain motions of increasing strain value. Each step strain was performed at a nominal strain rate of 20 s^−1^, followed by continuous monitoring of the cell deformation and force transducer signal for two minutes. The stage was then slowly returned to the starting position, followed by a 10 minute wait prior to executing the next step in the series.

### Calculating Strain

Because the nominal displacement of the stage does not necessarily correlate one-to-one with actual displacement of the contact lens surface, contact lens displacement was determined directly by imaging small 1 *µ*m beads embedded in the lens surface. A video was captured during each step using a high speed camera (Fastcam SA3, Photron, San Diego, CA), and analysis of the bead pixel position immediately before and after each step allowed determination of the top lens displacement, *l*. This information, combined with the gap distance, *d*, allowed calculation of the strain undergone in each step as 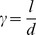. Typical strain values ranged from ∼0.1–3.0.

### Calculating Stress

A custom-made LabView (National Instruments, Austin, TX) program was used to record the force transducer output, *F*, during each step. In order to calculate the stress, the contact area between the cells and the contact lens, *A*, was needed. This value was estimated using ImageJ, [28] an image analysis software, to outline the cell contact area after top plate touchdown. Contact area varied with confluency but was typically around 70–90% of the total contact lens area exposed to the cell layer. Stress, 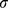, could then be calculated using the following equation: 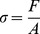. Typical stress values ranged from 10–350 Pa, depending on the applied strain.

### Calculating Relaxation Moduli

After each step-strain, the apparent relaxation modulus 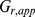 of the cell layer was calculated as 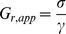. It is important that 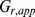 not be interpreted as a true relaxation modulus, such as that which would be measured for a complex fluid in continuous contact with the plates of a rheometer. This is because the measured stress is a complex superposition of the restoring forces of the cell bodies themselves and the number and strength of adherent contacts between the cells and the two surfaces onto which they are bound. Our observations of cells during straining deformations revealed cell detachment from the upper, contact lens surfaces and rarely from the lower plate. It is therefore more appropriate to consider that higher values of 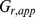 indicate greater force per area was required to achieve a particular strain, suggesting a greater number and/or strength of cell adhesions to the contact lens. Therefore, in the present study, the value of 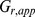 serves to provide an approximation of the relative extent of adhesion between the cells and the contact lens.

**Figure 2:**
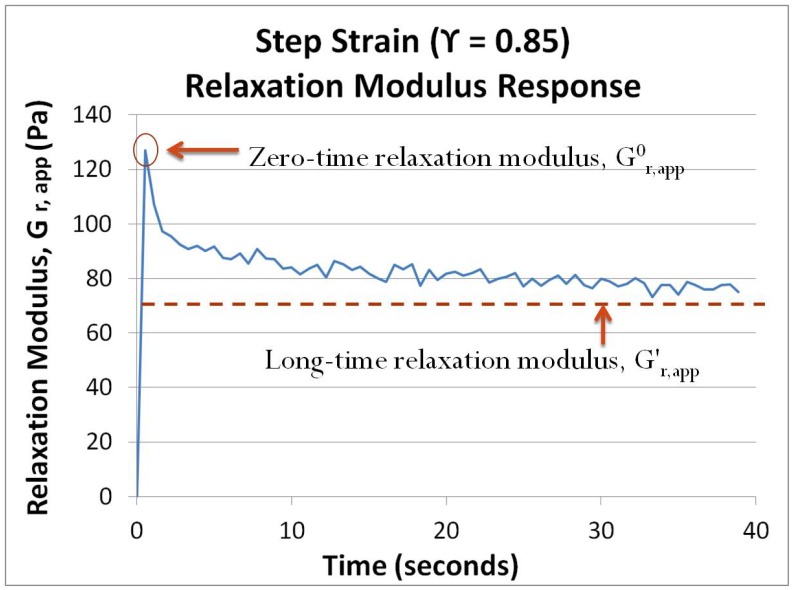
Example of a relaxation modulus curve for a given step strain experiment. The peak relaxation modulus recorded after the step, referred to as the zero-time relaxation modulus, 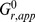, was used to compare the extent of cell adhesion between samples. The relaxation modulus relaxes to a new long-time value, 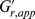. The value of 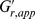 is greater than zero because cells are still in a strained state post-step.

The value of 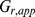 versus time was plotted for each step-strain. For conditions in which there was no cell attachment to the lens (for example, using a contact lens fresh from its packaging with no conditioning), the measured increase in stress (and therefore 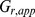) was very low. However, in cases where some cell attachment was evident (as confirmed by visualization during the step strain), the stress measurement rapidly rose to a maximum, followed by relaxation to a new equilibrium value due to the detachment of focal adhesions from the surface and re-organization within the cell monolayer. A characteristic curve in such a case is provided in Figure 2. The maximum value of 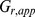 after each step, referred to as the zero-time relaxation modulus and designated as 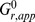, was used to compare the extent of cell attachment between samples, with higher values indicating a greater extent of cell attachment. The apparent relaxation modulus relaxes to a new long-time value, 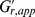. The value of 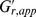 was consistently higher than zero, due to the sustained strain experienced by the cells that are still attached to both the top and bottom substrates.

### Bottom Substrate Preparation

The LCMR bottom trough consists of a metal dish with a small indentation that allows an 18×18 mm #2 coverslip (#060112-9, VWR, Radnor, PA) to sit securely in the base with the top surface of the coverslip just above the surface of the metal dish. Two days prior to each experiment, a coverslip was coated with a very low concentration of 1 *µ*m latex beads by applying 400 *µ*L of 0.00005 wt% 1 *µ*m diameter sulfate latex beads (Invitrogen #S37498, Life Technologies, Grand Island, NY) and allowing the solution to evaporate in a laminar flow hood overnight. The bead-coated coverslips were then affixed to the metal LCMR bottom dish with UV cured adhesive (Norland Optical Adhesive 61, Thorlabs, Newton, NJ) and coated with Cell Attachment Reagent (FNC coating mix C2605, US Biological, Salem, MA) at 0.2 mL/cm^2^. The bottom dish was then incubated at 37°C for at least 30 minutes, at which point any residual Cell Attachment Reagent was aspirated from the surface. The bottom dish was then ready to be seeded with cells.

### Cell Culture

Telemorase-immortalized human corneal epithelial cells (hTCEpi) [27] were maintained in KGM-2 Media (Clonetics C33107, Lonza, Basel, Switzerland), incubated at 37°C in 5%CO_2_ and passaged every 2 to 3 days. For testing, hTCEpi cells were detached from their culture dishes with TrypLE Express (Gibco 12605-036, Life Technologies) and seeded onto the mounted coverslips at a density of 1.5×10^4^ cell/cm^2^. This seeding density was chosen because it was found empirically to result in a 85–95% confluent layer of cells on the day of the experiment. The LCMR bottom dish was then maintained at 37°C in 5%CO_2_ for the following 48 hours. On the day of the experiment, the LCMR bottom dish was removed from the incubator and the regular culture media was exchanged for CO_2_-independent medium (Gibco C18045-088, Life Technologies). LCMR testing proceeded as described above.

In order to verify that the cells remained alive throughout the experiment, after randomly chosen experiments cells were detached from the bottom plate and top lens using TrypLE Express and exposed to 100% Trypan Blue 0.4% solution (T8154, Sigma Aldrich, St. Louis, MO) for 1 minute prior to being spun down in a centrifuge and resuspended in CO_2_-independent medium. Staining by Trypan Blue indicates cell structural damage and death. Relative to control dishes in which cells sat on the microscope in CO_2_-independent medium for two hours but were not exposed to an upper substrate, or control dishes that had remained in the incubator for two hours in KGM-2 growth media, none of the tested post-experiment samples showed an increase in Trypan Blue staining (≤0.5% of cells were positive for Trypan Blue for all conditions).

### Contact Lens Preparation

Contact lenses were pretreated in order to determine the effect of soaking solution and protein exposure on cell adhesion. Two silicon hydrogel lenses were used: PureVision balafilcon A (Bausch & Lomb, Rochester, NY) and AirOptix lotrafilcon B (Alcon, Fort Worth, TX). These two lenses were chosen for their popular use in the human patient population and in *in vitro* contact lens studies [29], [30], as well as their previously established propensity to accumulate lysozyme deposits in sufficient quantity to impact bacterial adhesion and health [6]. Three soaking solutions were tested: 1X PBS (Gibco 10010, Life Technologies), 1X BBS (B0231, Teknova, Hollister, CA) or commercially available Sensitive Eyes Plus Saline Solution (Bausch & Lomb). 1X PBS and 1X BBS were chosen because phosphate- and borate- based buffers are amongst the most common buffering systems used in commercial lens soaking solutions [31]. Sensitive Eyes Plus Saline Solution was chosen to represent a relatively simple commercially available solution which includes preservatives and salts in addition to buffering components. Sensitive Eyes Plus Saline Solution is an isotonic, borate-buffered solution containing boric acid, sodium borate, potassium chloride, and sodium chloride, as well as the preservatives polyaminopropyl biguanide (0.00003%) and edetate disodium (0.025%). Each soaking solution was used either pure (uncoated condition) or supplemented with lysozyme (L4919, Sigma Aldrich) or lactoferrin (L1294, Sigma Aldrich) protein at a concentration of 1 mg/mL. Lens treatment proceeded as follows: lenses were removed from their packaging and rinsed thoroughly with the solution they were to be soaked in. They were then submerged in a scintillation vial containing 15 mL soaking solution. Lenses remained submerged in solution at 25°C with gentle agitation for five days prior to testing, during which time any protein present in solution had the opportunity to deposit on the lens surface. The protein concentration and soaking time were chosen based on conditions previously shown to result in a substantial ≥1 *µ*g/lens lysozyme accumulation on both balafilcon A and lotrafilcon B lenses [17], [32]. Therefore, lenses incubated in pure soaking solution were considered to be uncoated controls, while lenses exposed to lysozyme- or lactoferrin-containing solutions were considered to be protein-coated. At least three duplicate experiments were run for each treatment condition; Table 1 details the number of experiments performed with each lens.

**Table 1:** Number of experiments run for each testing condition.

Lens Soaking Condition	PV (balafilcon A)	AO (lotrafilcon B)
	N =	N =
PBS	4	4
PBS + Lysozyme	3	3
BBS	3	3
BBS + Lysozyme	3	3
Sensitive Eyes	3	3
Sensitive Eyes + Lysozyme	6	3
Sensitive Eyes + Lactoferrin	4	-

Table showing the number (N) of experiments run with each condition for both contact lens types. The effect of lactoferrin was only tested to provide a general comparison to the lysozyme response, and therefore was only tested in combination with Sensitive Eyes solution on PureVision lenses rather than the full panel of conditions.

Immediately prior to the experiment, each contact lens was gently stamped onto a coverslip covered with a low concentration of 1 *µ*m beads, causing a small number of these beads to adhere to the contact lens surface. These beads were used in the experiment to calculate the gap distance between the lens and the bottom plate and to track the position of the top lens. All lenses were then briefly rinsed with pure soaking solution prior to mounting them onto the upper holder, which utilized a metal brace to gently hold the lens against a slightly curved glass surface.

## Results

**Figure 3:**
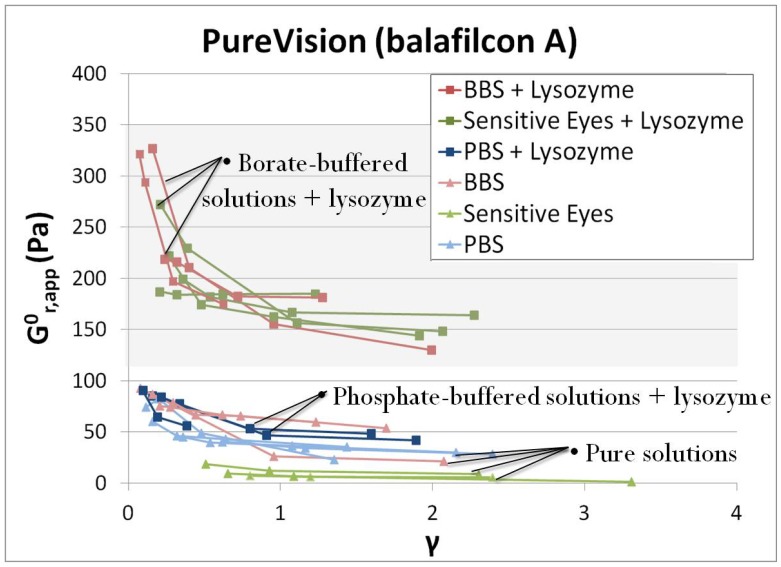
Zero-time relaxation modulus, 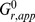 versus strain data for PV lenses. Each line represents data taken from a single cell monolayer, with each point representing a single step strain measurement.

Figure 3 shows results of testing done on PV contact lenses. The results are presented in the form of the zero-time relaxation modulus, 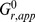, of the epithelial cell monolayer as a function of applied strain. For a given monolayer, 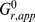 was observed to strain soften, or decrease as higher strains were tested. This softening is characteristic of a system in which fragile adhesions are being broken at lower strains that do not re-form before higher strains are tested. This suggests that some adhesive contacts between the cells and the contact lenses are detached with strain; this was confirmed by visual observation during the step strain experiments.

**Figure 4:**
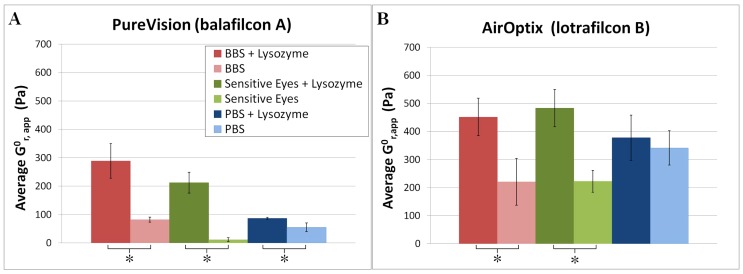
Histograms showing the average zero-time relaxation modulus for the first step performed on each monolayer with PV (A) and AO (B) lenses. Error bars represent standard deviation. For each individual soaking solution (PBS, BBS, and Sensitive Eyes), a two-tailed Student's *t*-test was used to compare the uncoated and protein-coated condition. Asterisk (*) signifies that there is a statistically significant (*p*≤0.05) difference between the two conditions.

As shown in Figure 3, exposure to lysozyme increased 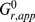 for all soaking solutions. This increase was greatest for lysozyme in the two borate-buffered solutions, BBS and Sensitive Eyes, with 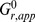 values up to 3-fold greater than that of the uncoated samples. Figure 4 presents the PV lens data in a histogram format (Figure 4A), as well as data for AO lenses in Figure 4B. Figure 4 compares the average value of 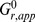 for the first step strain performed on each monolayer. Only the first step strain performed on each monolayer was used in calculating this average, in order to avoid including the effects of permanent cell detachment and subsequent strain softening in later step strains. The data was also analyzed by averaging the moduli for every strain performed, as well as by comparing average moduli for similar strain values, all of which preserved the ordering and significant differences between samples provided in Figure 4. The results of these analyses are provided in Figures S1 and S2.

Figure 4 shows that for both lens types, exposure to lysozyme in borate-buffered solutions greatly increased the adhesion of corneal cells to the lens (an over 3-fold increase for PV lenses, 2-fold increase for AO lenses). An increase was also seen for exposure to lysozyme in the phospate-buffered solution PBS, but to a much lesser extent (60% increase for PV lenses, no statistically significant increase for AO lenses).

### Effect of Lens Type

As seen in Figure 4, the value of 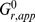 also depended a great deal on lens type. Relative to the PV lenses, AO lenses had higher relaxation moduli overall, including for the case of uncoated lenses soaked in pure solutions. AO lenses soaked in pure solutions had average 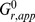 values at least 160% higher than PV lenses under the same conditions. A similar trend was observed for the protein-coated lenses: lysozyme coated AO lenses had at least 50% higher 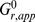 values than the matched-condition PV lenses.

### Effect of Lactoferrin

**Figure 5:**
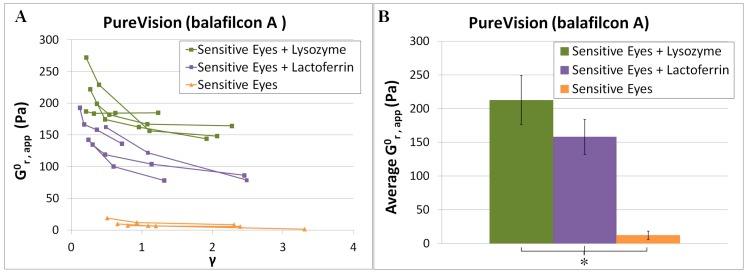
Zero-time relaxation modulus versus strain data for PV lenses soaked in pure Sensitive Eyes solution versus Sensitive Eyes with lactoferrin or lysozyme. A: Each line represents data taken from a single cell monolayer, with each point representing a single step strain measurement. B: Histogram comparing the average zero-time modulus for the first step performed on each monolayer. Error bars represent standard deviation. Each condition was compared individually to the other two conditions using a two-tailed Student's *t*-test. Asterisk (*) signifies a statistically significant (*p*≤0.05) difference between the conditions.

In order to determine whether an increase in cell adhesion would be observed for other common tear film proteins such as lactoferrin, PV lenses were also tested with lactoferrin in Sensitive Eyes. As shown in Figure 5, lactoferrin in sensitive eyes increased cell attachment relative to an uncoated lens from 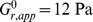 to 
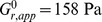
, a 13-fold increase. However, this increase was slightly less than the 17-fold increase observed with lysozyme. Lactoferrin was tested in this study solely to establish whether cell adhesion might be enhanced by lens exposure to other proteins in addition to lysozyme. Therefore, a full set of data testing lactoferrin combined with other soaking solutions, or with AO lenses, was not collected. The effect of lactoferrin deposition under the full set of buffer and lens conditions, as well as the impact of other tear film components such as mucin and meibum, will be the subject of future studies.

## Discussion

For both contact lens materials studied, the presence of lysozyme in the soaking solution increased the extent of cell adhesion, regardless of the soaking buffer used. This observation held true for both lysozyme and lactoferrin proteins, which are commonly found in the conditioning films that form during extended contact lens wear. This increase in adhesion was likely due to the adsorbed protein offering favorable sites for formation of focal adhesions, possibly via a similar mechanism to that proposed for bacterial adhesion in which exposed carbohydrate moieties serve as binding sites [6]–[8]. These findings demonstrate that in addition to affecting bacterial attachment, the conditioning film may also have an effect on the tendency of the corneal epithelial layer to form intermittent attachments with the lens surface. When combined with studies indicating increased epithelial barrier permeability after extended wear of soft contact lenses, [12], [21], [22] this observation argues for the potential existence of a complex mechanical relationship between the contact lens surface and the corneal epithelium that is deserving of further study. Future work needs to be done to determine what, if any, impact such attachments may have on lens comfort or other clinical outcomes.

One particularly intriguing finding is that although lysozyme increased cell adhesion in all of the solutions tested, it did so to a much greater extent in the borate-buffered solutions (BBS and Sensitive Eyes) relative to phosphate-buffered saline (PBS). The root cause of this difference is not addressed in this study, but it seems likely that the buffer identity influences the conformation of the dissolved protein in a way that either (1) induces more protein to deposit onto the contact lens surface or (2) exposes more adhesion-promoting sites for cell attachment after protein is deposited. It is also possible that both of these mechanisms contribute simultaneously to the observed increase in cell attachment. The implication is that care should be taken in choosing the buffering components in lens care solutions in which lenses may soak in the presence of tear film proteins, as these may influence the propensity of the lens to interact mechanically with the cell layer.

Even though corneal cell adhesion to both AO and PV lenses increased in a buffer-dependent manner upon exposure to lysozyme, the absolute values of 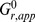 differed greatly between the two lens types. This was true even for uncoated lenses soaked in pure buffer: AO lenses soaked in pure Sensitive Eyes solution exhibited 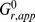 values 18-fold higher than PV lenses under the same conditions. Similarly, AO lenses soaked in pure PBS and pure BBS had 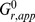 values 6.2-fold and 2.7-fold higher than those for matched PV lenses. Thus while the soaking solution and protein coating have a strong impact on corneal cell attachment, the basic structure and surface chemistry of each lens type also plays a significant role. This is not an entirely surprising result; it has been observed previously that the surface chemistry of synthetic materials modulates a variety of cellular functions, including cell adhesion, proliferation, and differentiation [33], [34]. This influence arises from a variety of factors, including the chemical identity of exposed moieties at the surface, hydrophobicity, and charge [33]. Thus the distinct hydrogel chemistry and surface plasma treatments of AO and PV lenses may lead to an innate variation in the tendency of corneal cells to attach to the different contact lens types, even in the absence of a protein coating.

There were also subtle differences in the behavior of the two lenses when protein was added to the soaking solution. Although the general trend of higher 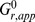 values for lysozyme-exposed lenses was observed for both lens types, AO lenses were less strongly effected. For example, AO lenses exposed to lysozyme in either Sensitive Eyes or BBS had 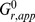 values ∼50% higher than AO lenses soaked in pure solutions. However, PV lenses exposed to lysozyme in either Sensitive Eyes or BBS gave 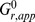 values that were 3-fold and 18-fold greater, respectively, than uncoated PV lenses soaked in those buffers. Previous studies have demonstrated that the nature of the conditioning film that forms on lenses differs based on the lens chemistry [18], [19]. In addition, cell adhesion onto synthetic surfaces coated with proteins has been shown to depend a great deal on the surface structure, which controls integrin-mediated adhesion via the conformation of the adsorbed species [33]–[35]. Therefore the differences in 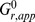 observed for protein-exposed AO and PV lenses under identical conditions likely arise from differences in adhered protein concentration and conformation, both of which are in turn influenced by the chemistry of the lens surface itself.

It is important to note that the results reported here were collected for a corneal epithelial cell monolayer exposed to cell culture medium, as opposed to an artificial tear film. *In vivo*, the corneal surface is lubricated by a tear film containing several components besides lysozyme and lactoferrin, including meibum and several lipids [13]. It is possible that in the presence of these additional factors, corneal cell interaction with the uncoated and protein-coated lenses may differ, and these components may also deposit on the lens during the two-hour period of lens contact with the cells [36]. The CO_2_-independent medium used in this initial study does not contain any artificial buffers and was not supplemented with serum. Thus, the present study serves as a “base case” for testing corneal cell adhesion with a cell culture medium that is known to maintain cell viability, uses biocompatible buffers, and contains neither exogenous serum proteins nor additional tear film components such as meibum or lipids. Future experiments with the LCMR may utilize a more complex artificial tear film model including the lipids, waxes, and proteins present in the tear film.

This study employed a modified cell monolayer rheometer to determine the impact of lens-deposited tear film proteins on corneal epithelial cell adhesion to soft contact lens hydrogels. Significantly, these measurements were conducted while subjecting the cells to moderate surface pressures and shear rates, similar to those experienced by corneal cells *in vivo*. It should be noted that the LCMR system described above currently does not allow the testing of cell multilayers, and the corneal epithelial monolayers tested in this study are not identical to the apical cell layer of a fully differentiated multilayer corneal model. Nevertheless, the response of corneal epithelial cell monolayers in this experiment suggests a strong influence of tear film deposition on cell interactions with the contact lens surface. Furthermore, this effect is mediated by the nature of the buffer in which the tear film proteins are dissolved. In the future it may be possible to perform similar tests on a more complex multilayer corneal model using a broader variety of lenses and treatment protocols.

## Supporting Information

Figure S1 **Histograms showing the average zero-time relaxation modulus for strain values between 0.05 and 0.2 for step strains performed on each monolayer with PV (A) and AO (B) lenses.** Error bars represent standard deviation. For each individual soaking solution (PBS, BBS, and Sensitive Eyes), a two-tailed Student's *t*-test was used to compare the uncoated and protein-coated condition. Asterisk (*) signifies that there is a statistically significant (*p*≤0.05) difference between the two conditions.
(TIF)

Figure S2 **Histograms showing the average zero-time relaxation modulus for all step strains performed on each monolayer with PV (A) and AO (B) lenses.** Error bars represent standard deviation. For each individual soaking solution (PBS, BBS, and Sensitive Eyes), a two-tailed Student's *t*-test was used to compare the uncoated and protein-coated condition. Asterisk (*) signifies that there is a statistically significant (*p*≤0.05) difference between the two conditions.
(TIF)

## References

[1] Rosenfeld L, Cerretani C, Leiske DL, Toney MF, Radke CJ, et al (2013) Structural and Rheological Properties of Meibomian Lipid. Invest Ophthalmol Vis Sci 54: 2720–2732. 23513065 10.1167/iovs.12-10987

[2] Leiske DL, Raju SR, Ketelson HA, Millar TJ, Fuller GG (2010) The interfacial viscoelastic properties and structures of human and animal Meibomian lipids. Exp Eye Res 90: 598–604. 20156438 10.1016/j.exer.2010.02.004

[3] Flanagan JL, Willcox MDP (2009) Role of lactoferrin in the tear film. Biochimie 91: 35–43. 18718499 10.1016/j.biochi.2008.07.007

[4] Williams TJ, Schneider RP, Willcox MDP (2003) The effect of protein-coated contact lenses on the adhesion and viability of gram negative bacteria. Curr Eye Res 27: 227–35. 14562174 10.1076/ceyr.27.4.227.16602

[5] Willcox MDP, Harmis N, Cowell BA, Williams T, Holden BA (2001) Bacterial interactions with contact lenses; effects of lens material, lens wear and microbial physiology. Biomaterials 22: 3235–3247. 11700795 10.1016/s0142-9612(01)00161-2

[6] Subbaraman LN, Borazjani R, Zhu H, Zhao Z, Jones L (2011) Influence of Protein Deposition on Bacterial Adhesion to Contact Lenses. Optom Vis Sci 88: 959–966. 21602733 10.1097/OPX.0b013e31821ffccb

[7] Klotz SA, Misra RP, Butrus SI (1987) Carbohydrate deposits on the surfaces of worn extended-wear soft contact lenses. Arch Ophthalmol 105: 974–7. 3475046 10.1001/archopht.1987.01060070118039

[8] Tripathi PC, Tripathi RC (1992) Analysis of glycoprotein deposits on disposable soft contact lenses. Invest Ophthalmol Vis Sci 33: 121–5. 1730532

[9] Sack RA, Jones B, Antignani A, Libow R, Harvey H (1987) Specificity and biological activity of the protein deposited on the hydrogel surface. Relationship of polymer structure to biofilm formation. Invest Ophthalmol Vis Sci 28: 842–9. 3570694

[10] Ng A, Heynen M, Luensmann D, Subbaraman LN, Jones L (2013) Impact of tear film components on the conformational state of lysozyme deposited on contact lenses. J Biomed Mater Res B Appl Biomater: 1–10. 10.1002/jbm.b.32927 23564739

[11] Lin MC, Soliman GN, Song MJ, Smith JP, Lin CT, et al (2003) Soft contact lens extended wear affects corneal epithelial permeability: hypoxic or mechanical etiology. Cont Lens Anterior Eye 26: 11–6. 16303492 10.1016/S1367-0484(02)00088-7

[12] Duench S, Sorbara L, Keir N, Simpson T, Jones L (2013) Impact of Silicone Hydrogel Lenses and Solutions on Corneal Epithelial Permeability. Optom Vis Sci 90: 546–556. 23645374 10.1097/OPX.0b013e318294c2a6

[13] Sack RA, Tan KO, Tan A (1992) Diurnal tear cycle: evidence for a nocturnal inammatory constitutive tear fluid. Invest Ophthalmol Vis Sci 33: 626–640. 1544788

[14] Leahy CD, Mandell RB, Lin ST (1990) Initial in vivo tear protein deposition on individual hydrogel contact lenses. Optom Vis Sci 67: 504–511. 2205814 10.1097/00006324-199007000-00008

[15] Lin ST, Mandell RB, Leahy CD, Newell JO (1991) Protein accumulation on disposable extended wear lenses. CLAO J Off Publ Contact Lens Assoc Opthamologists 17: 44–50. 2007285

[16] Jones L, Optom FC, Senchyna M, Glasier M, Schickler J, et al (2003) Lysozyme and Lipid Deposition on Silicone Hydrogel Contact Lens Materials. Eye Contact Lens 29: 75–79. 10.1097/00140068-200301001-00021 12772737

[17] Subbaraman LN, Glasier MA, Senchyna M, Sheardown H, Jones L (2006) Kinetics of in vitro lysozyme deposition on silicone hydrogel, PMMA, and FDA groups I, II, and IV contact lens materials. Curr Eye Res 31: 787–796. 17050272 10.1080/02713680600888799

[18] Soltys-Robitaille CE, Ammon DM, Valint PL, Grobe GL (2001) The relationship between contact lens surface charge and in-vitro protein deposition levels. Biomaterials 22: 3257–60. 11700797 10.1016/s0142-9612(01)00163-6

[19] Garrett Q, Garrett RW, Milthorpe BK (1999) Lysozyme sorption in hydrogel contact lenses. Invest Ophthalmol Vis Sci 40: 897–903. 10102286

[20] McNamara NA, Polse KA, Fukunaga SA, Maebori JS, Suzuki RM (1998) Soft Lens Extended Wear Affects Epithelial Barrier Function. Ophthalmology 105: 2330–2335. 9855168 10.1016/S0161-6420(98)91237-4

[21] Andrasko G, Ryen K (2008) Corneal staining and comfort observed with traditional and silicone hydrogel lenses and multipurpose solution combinations. Optometry 79: 444–54. 18656083 10.1016/j.optm.2008.04.097

[22] Jones L, MacDougall N, Sorbara G (2002) Asymptomatic Corneal Staining Associated with the Use of Balafilcon Silicone-Hydrogel Contact Lenses Disinfected with a Polyaminopropyl. Optom Vis Sci 79: 753–761. 12512683 10.1097/00006324-200212000-00007

[23] Dunn AC, Cobb JA, Kantzios AN, Lee SJ, Sarntinoranont M, et al (2008) Friction Coefficient Measurement of Hydrogel Materials on Living Epithelial Cells. Tribol Lett 30: 13–19.

[24] Conway HD, Richman MW (1983) The effects of contact lens deformation on tear film pressure and thickness during motion of the lens towards the eye. J Biomech Eng 105: 47–50. 6843101 10.1115/1.3138383

[25] Martin DK, Holden BA (1986) Forces developed beneath hydrogel contact lenses due to squeeze pressure. Phys Med Biol 31: 635–49. 3749253 10.1088/0031-9155/31/6/005

[26] Nairn JA, Jiang T (1995) Measurement of the friction and lubricity properties of contact lenses. Proc ANTEC Annu Tech Conf 6: 1–5.

[27] Robertson DM, Li L, Fisher S, Pearce VP, Shay JW, et al (2005) Characterization of growth and differentiation in a telomerase-immortalized human corneal epithelial cell line. Invest Ophthalmol Vis Sci 46: 470–8. 15671271 10.1167/iovs.04-0528

[28] Abramoff MD, Magalhaes PJ, Ram Sunanda JR (2004) Image Processing with ImageJ. Biophotonics Int 11: 36–42.

[29] Suwala M, Glasier MA, Subbaraman LN, Jones L (2007) Quantity and conformation of lysozyme deposited on conventional and silicone hydrogel contact lens materials using an in vitro model. Eye Contact Lens 33: 138–43. 17502748 10.1097/01.icl.0000244155.87409.f6

[30] Pucker AD, Thangavelu M, Nichols JJ (2010) In vitro lipid deposition on hydrogel and silicone hydrogel contact lenses. Invest Ophthalmol Vis Sci 51: 6334–40. 20702829 10.1167/iovs.10-5836

[31] White P (2012) 2012 Contact Lenses & Solutions. Contact Lens Spectr: 1–28.

[32] Ng A, Heynen M, Luensmann D, Jones L (2012) Impact of Tear Film Components on Lysozyme Deposition to Contact Lenses. Optom Vis Sci 89: 392–400. 22388670 10.1097/OPX.0b013e31824c0c4a

[33] Keselowsky BG, Collard DM, Garcia AJ, Weinbaum S (2013) Integrin surface binding chemistry specificity regulates biomaterial on effects cell differentiation. PNAS 102: 5953–5957. 10.1073/pnas.0407356102 PMC1087905 15827122

[34] Keselowsky BG, Collard DM, García AJ (2004) Surface chemistry modulates focal adhesion composition and signaling through changes in integrin binding. Biomaterials 25: 5947–54. 15183609 10.1016/j.biomaterials.2004.01.062

[35] Keselowsky BG, Collard DM, García AJ (2003) Surface chemistry modulates fibronectin conformation and directs integrin binding and specificity to control cell adhesion. J Biomed Mater Res A 66: 247–59. 12888994 10.1002/jbm.a.10537

[36] Andrade JD, Hlady V (1986) Protein Adsorption and Materials Biocompatibility: A Tutorial Review and Suggested Hypotheses. In: Adv. Polym. Sci., Berlin: Springer-Verlag. pp. 3–58.

