# Supplementary figures and images for: Corneal Cell Adhesion to Contact Lens Hydrogel Materials Enhanced via Tear Film Protein Deposition
Source: PLoS One. 2014 Aug 21;9(8):e105512. doi: 10.1371/journal.pone.0105512 (PMC4140805; doi:10.1371/journal.pone.0105512)

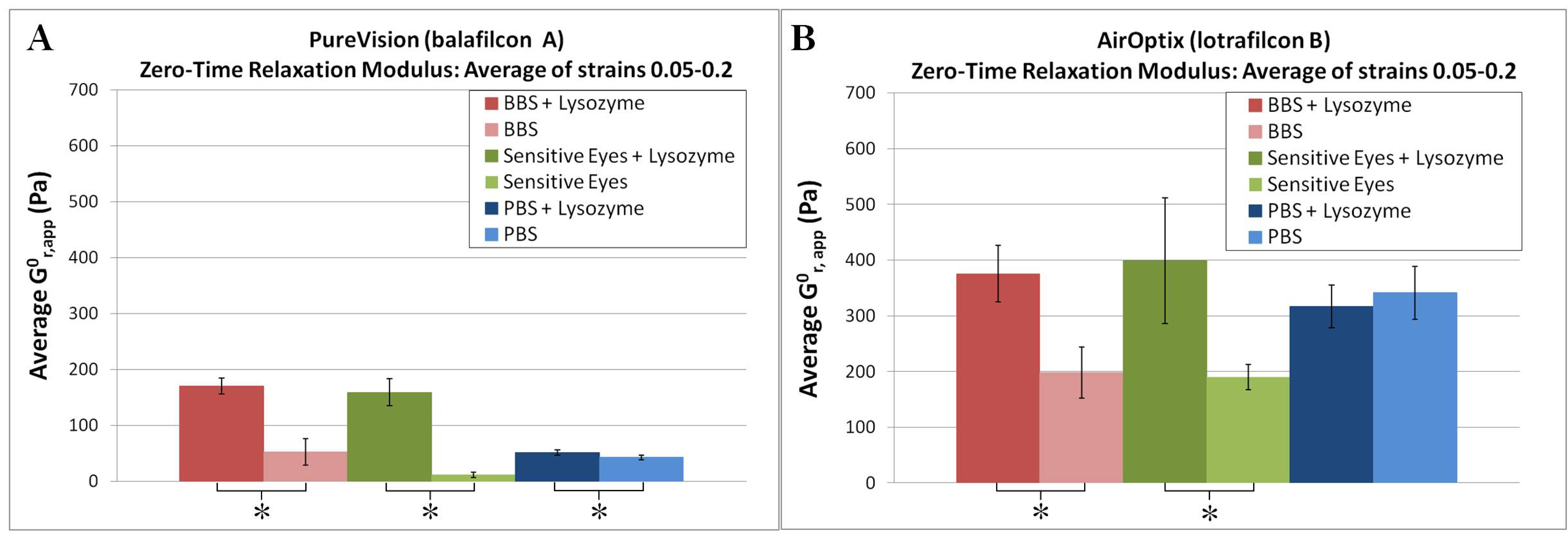

Supplement: Figure S1 — Histograms showing the average zero-time relaxation modulus for strain values between 0.05 and 0.2 for step strains performed on each monolayer with PV (A) and AO (B) lenses. Error bars represent standard deviation. For each individual soaking solution (PBS, BBS, and Sensitive Eyes), a two-tailed Student's t-test was used to compare the uncoated and protein-coated condition. Asterisk (*) signifies that there is a statistically significant (p≤0.05) difference between the two conditions. (TIF) [file pone.0105512.s001.tif]

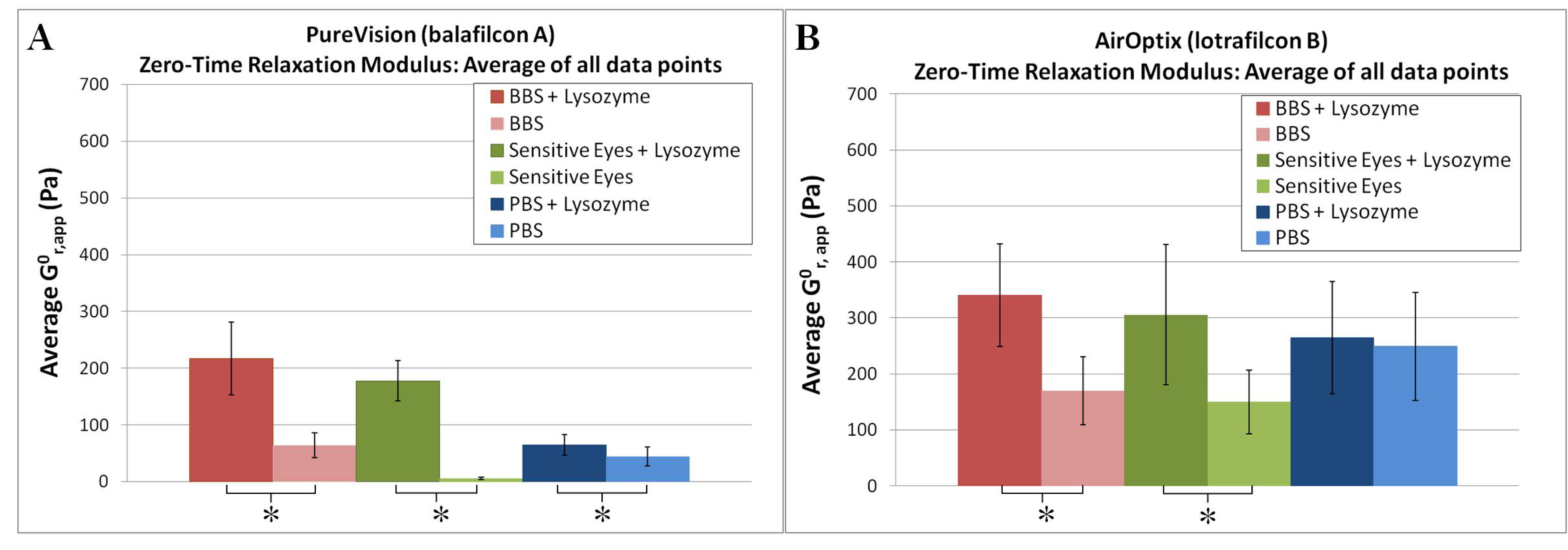

Supplement: Figure S2 — Histograms showing the average zero-time relaxation modulus for all step strains performed on each monolayer with PV (A) and AO (B) lenses. Error bars represent standard deviation. For each individual soaking solution (PBS, BBS, and Sensitive Eyes), a two-tailed Student's t-test was used to compare the uncoated and protein-coated condition. Asterisk (*) signifies that there is a statistically significant (p≤0.05) difference between the two conditions. (TIF) [file pone.0105512.s002.tif]
